# 21st century agriculture: integration of plant microbiomes for improved crop production and food security

**DOI:** 10.1111/1751-7915.12180

**Published:** 2014-12-27

**Authors:** Angela Sessitsch, Birgit Mitter

**Affiliations:** Health & Environment Department, Bioresources Unit, AIT Austrian Institute of Technology GmbHKonrad-Lorenz-straße 24, 3430, Tulln, Austria

Similar to the human gut are plants and in particular plant roots tightly associated with complex microbial communities. Microbiomes of both, gut and plants, are known for their importance for the host's nutrient uptake, protection against pathogens and abiotic stress as well as for providing metabolic capacities (Sekirov *et al*., 2010; Mitter *et al*., 2013; Ramírez-Puebla *et al*., 2013). The plant microbiome has been further suggested as an extension of the host phenotype (Aleklett and Hart, 2013). Plant–microbe interactions are highly specific with plant microbiota being driven by the host genotype and physiology (e.g. root exudates and metabolites) as well as environmental factors (Rasche *et al*., 2006; Lundberg *et al*., 2012). Few examples of beneficial plant–microbe interactions are well investigated and explored in regard to their importance in agricultural systems. These include biological nitrogen fixation by rhizobia, which establish a symbiosis with legumes and represent the basis of crop rotations including legumes contributing to the maintenance of soil fertility. Furthermore, about 80% of land plant species are internally colonized by arbuscular mycorrhizal fungi. In this symbiosis, arbuscules and vesicles are formed from the hyphae being particularly important for plant nutrient acquisition. In addition, the more specialized symbiotic defensive mutualism between Pööideae grasses and endophytic fungi of the *Epichloë* is well explored (Clay, 1988) and important for pasture production. Apart from these well-known mutualistic plant–microbe interactions, beneficial microorganisms have been hardly considered in crop production strategies. However, considering the demonstrated functional importance of the plant microbiome, the effects that can be observed upon the inoculation of selected microorganisms and the fact that plants and microorganisms carry genetic determinants needed for their interaction, we predict that plant microbiome functions will be an essential component of tomorrow's crop production.

Plant microbiome composition is affected by various host-driven factors, including for instance the plant genotype, and by agricultural practices such as fertilization or pesticide application. Although we still hardly understand how microbiome functioning is affected by such structural changes, it is likely that functioning will be affected as well. Whereas conventional agriculture has not yet started to consider potential harm on the functioning of plant-associated microbiota due to current practices, organic farming systems generally aim at making best use of natural resources and maintaining biodiversity (Mader *et al*., 2002). For instance, crop rotations with legumes are applied, and usually higher plant diversity is used or maintained resulting in a more efficient exploration and maintenance of microbial functions. Alongside the general trend to increase the sustainability of agricultural practices such as different soil preparation practices, fertilizer or pesticide treatments might be better selected in regard to favouring or exhibiting least adverse effects on desirable plant microbiome functions. Apart from efficacy, the effect on the plant microbiome could be one selection criterion. Furthermore, dosage effects might be important to consider. Overdosing fertilizers or pesticides might have more adverse effects on microbiome activities than lower amounts still suitable for suitable efficacy.

Industry has started to exploit individual microorganisms mostly as microbial plant protection products or as biofertilizers. There is a rapidly increasing interest from the industry on microbial products due to a far higher demand of alternatives to current pesticides and fertilizers strongly promoted by national strategic plans to restrict chemical input in agriculture. However, despite the high potential such microbial inoculants have frequently shown in lab and greenhouse experiments, the efficacy and the consistency of desired effects of microorganisms under various field conditions still represent a major bottleneck for product development. Therefore, there is an urgent need to improve selection processes, application techniques and particularly to better understand the interaction between plants and microorganisms under field conditions. Tremendous information on the mechanisms involved in plant–microbe interactions has been obtained with model plants grown under gnotobiotic conditions. Now, scientists have started to realize that for improving field efficiency, it is of utmost importance to use relevant plant cultivars and to better understand microbial activities in the field. Such an understanding will reveal under which (field-relevant) conditions a microbial strain exhibits desired activities, or whether co-colonizing microorganisms promote or interfere with specific activities of an inoculant strain. This in combination with improved application technologies and improved formulations will greatly improve the efficacy of microbial products. In the future, we will also be able to make better use of synergistic and complementary mechanisms of individual strains and design microbiomes supporting plant growth and health. Similarly, concepts might be developed considering the transplantation of microbiomes, for instance plant microbiomes growing well under adverse conditions could serve as inoculants or at least as model for the design of ‘synthetic’ microbiomes.

Plants respond to microbiota, have genetic determinants to interact with microorganisms, and relationships between host and microbiome evolution have been shown (Bouffaud *et al*., 2014; Delaux *et al*., 2014). This implies that plants could be improved either by genetic improvement, selection or breeding in regard to a more efficient interaction with beneficial microorganisms. Whereas in the last decades, plants have been mostly improved and selected for higher yield and resistance, gazing into our crystal ball we foresee that efficient interaction with beneficial microorganisms will be an additional breeding target. Applications might range from breeding legumes for improved interaction with well-known rhizobial symbionts to crops reducing interactions with specific pathogens and enhancing mutualists or plants triggering specific microbiome components. A more detailed understanding on the molecular mechanisms used by plants to interact with mutualists will lead to the development of suitable breeding targets and screening approaches. Plant breeding or genetic modification may lead in the future to the identification of plant lines ensuring improved resource efficiency, tolerance of abiotic stress and defense against pests and pathogens.

Maintaining plant beneficial microbiome functions is particularly important for maintaining yield stability and to enable plant growth under (sometimes unexpected) suboptimal conditions such as drought or pathogen infestation. Although plant microbiomes have high potential to improve overall crop production worldwide, they will be particularly important for plant production under constrained conditions, where limited resources are available to irrigate, fertilize or treat plant diseases. This is the case in many parts of the world, where low input agriculture is common practice and improved germ plasm or agricultural amendments are hardly available. Making better use of plant microbiome functions will particularly support agricultural production under these conditions and foster the bio-economy of less developed countries providing microbial inoculants and establishing strain collections from local environments.

## Conflict of interest

Authors have no conflict of interest to declare.
